# Scorpio and centipede ameliorate asthma by inhibiting the crosstalk between ferroptosis and inflammation in airway epithelial cells

**DOI:** 10.22038/IJBMS.2023.70411.15306

**Published:** 2023

**Authors:** Yada Zhang, Yiren Chen, Yingen Wu, Ling Yang, Hong Fang, Yanqi Cheng, Yuqin Wu, Binqing Tang

**Affiliations:** 1 Department of Respiratory Medicine, Shanghai Municipal Hospital of Traditional Chinese Medicine, Shanghai University of Traditional Chinese Medicine, Shanghai, China; 2 Department of Internal Medicine, Longhua Hospital, Shanghai University of Traditional Chinese Medicine, Shanghai, China; 3 Department of Geriatrics, Shanghai Fourth People’s Hospital Affiliated to Tongji University, Shanghai, China; 4 Prevention and Health Care Department of TCM, Longhua Hospital, Shanghai University of Traditional Chinese Medicine, Shanghai, China; # These authors contributed eqully to this work

**Keywords:** Airway epithelial cells, Asthma, Ferroptosis, Inflammation, Scorpio and centipede

## Abstract

**Objective(s):**

We previously revealed that scorpio and centipede (SC) improve the inflammatory response in asthma, whereas it is unclear whether ferroptosis is involved in this process.

**Materials and Methods:**

The asthmatic mouse model was established and lung tissues were collected for histopathological examination. The levels of tumor necrosis factor-α (TNF-α), interleukin- (IL-)1β, Fe^2+^, malondialdehyde (MDA), glutathione peroxidase 4 (GPX4), ferritin heavy chain 1(FTH1), and reactive oxygen species (ROS) were assessed in asthmatic mice and mouse airway epithelial cells.

**Results:**

Our results showed that ferroptosis was induced in asthmatic mice, as evidenced by the reduction of FTH1 and GPX4 expression and the increase of MDA and Fe^2+^ levels (all *P*<0.05). Ferrostatin-1 repressed inflammation and ferroptosis of asthmatic mice. Additionally, SC significantly inhibited the levels of TNF-α, IL-1β, MDA, and Fe^2+^, while enhancing FTH1 and GPX4 expression. However, SC plus erastin showed the reverse results. Moreover, ferroptosis remarkably increased in asthmatic airway epithelial cells, while SC suppressed ferroptosis of the cells (all *P*<0.05).

**Conclusion:**

SC ameliorated asthma by inhibiting the crosstalk between ferroptosis and inflammation in airway epithelial cells.

## Introduction

Asthma is a chronic inflammatory airway disease, which is characterized by inflammation, airway hyperresponsiveness, and airway remodeling (1-3). Asthma threatens the health and safety of approximately 300 million people all over the world (4). The pathological manifestations of asthma show the dysfunction of the lung airway system caused by the airway epithelium hyperplasia, mucosal swelling, and increased bronchial secretions, and the clinical features are cough, chest distress, shortness of breath, and dyspnea (5). The drug treatment of asthma includes potent anti-inflammatory drugs, inhaled corticosteroids, antiallergic, rapid-acting bronchodilators, and others at the moment (6, 7). In the progress of asthma, immune cells infiltrate into the airways, and these cells activate the production of mast cells and immunoglobulin E by secreting the Th2 cytokines, which subsequently leads to the clinical features of asthma (8). Therefore, inhibiting the inflammation of the respiratory system may be a promising strategy in asthma.

Ferroptosis is an iron-dependent, p53-mediated nonapoptotic cell death. The occurrence of ferroptosis is characterized by accumulation of reactive oxygen species (ROS), glutathione (GSH) depletion, and reduced glutathione peroxidase 4 (GPX4) activity (9). Recently, studies demonstrated that ferroptosis promoted the inflammatory response in cells, which may be a novel therapeutic target in inflammation-related diseases (10, 11). Arachidonic acid metabolism is one of the mechanisms by which ferroptosis regulates inflammation. Recent research suggests that ferroptosis can directly enhance PTGS2 expression, facilitate the metabolism of arachidonic acid, and contribute to the secretion of inflammatory factors (12). In addition, GPX4 plays a cytoprotective role by repressing the levels of cellular lipid hydroperoxides, which promote inflammation (13). Moreover, ferroptosis promotes the inflammatory response by producing cytokines, such as interleukin-(IL)-13 (14). The expression levels of pro-inflammatory cytokines, including tumor necrosis factor-α (TNF-α), IL-6, and IL-1β, were significantly decreased in the non-alcoholic steatohepatitis by inhibiting ferroptosis (15). Ferrostatin-1 (Fer-1) is a ferroptosis-specific inhibitor, which alleviates osteoarthritis by decreasing inflammation and rescuing the expression of collagen II (16). Inhibiting ferroptosis can relieve the inflammatory response in ulcerative colitis by regulating the Nrf2/HO-1 signaling pathway (17). Additionally, ferroptosis was discovered to participate in the progression of asthma. Bao *et al*. found that liproxstatin-1 mitigated neutrophilic asthma via repressing ferroptosis (18). Another study uncovered that house dust mites triggered airway epithelial cell ferroptosis in asthma (19). However, whether the crosstalk between ferroptosis and inflammation is involved in asthma is indistinct.

Scorpio and centipede (SC) are traditional Chinese medicines and have been used in asthma, stroke, arthritis, and other diseases due to anti-inflammation and dredging collaterals (20-22). SC contribute to the improvement of inflammation and remodeling of the airway in asthmatic rats. In our previous study, we found that SC improved the clinical symptom, ameliorated the function of the lung, and reduced the inflammation of the airway in 78 cases of refractory asthma (23). In light of the background above, we wonder whether SC ameliorates the function of airway epithelial cells by influencing the crosstalk between ferroptosis and inflammation.

In the present study, we investigated the effect and mechanism of SC in asthma by studying ferroptosis and inflammation. First, we established the mouse model of asthma, and we observed the pathological changes in the lung tissues and analyzed the levels of pro-inflammatory cytokines, iron accumulation, and ferroptosis-related genes. *In vitro*, iron accumulation and ferroptosis-related genes were detected in airway epithelial cells stimulated with cytokines treated with SC.

## Materials and Methods

***Experimental animals***

The BALB/c mice (6–8 weeks, 20 ± 2g) were purchased from Shanghai Laboratory Animal Center (Shanghai, China). The mice were kept in a specified pathogen-free environment (12 hr light/dark cycle, 24 °C± 2 °C, 50%–70% humidity), with food and water freely. Our experiments are approved by the Ethics Committee of Animal Experiments of Shanghai Municipal Hospital of Traditional Chinese Medicine, Shanghai University of Traditional Chinese Medicine, China.

***Asthma mouse model***

To study the improvement effect of ferroptosis on asthma, the male BALB/c mice were randomly allocated to three groups (n = 3), including the control group, asthma group, and Fer-1 group. To investigate whether SC improves asthma through ferroptosis, mice were divided into three other groups (n = 3), including asthma, SC, and SC+erastin. Ovalbumin (OVA) was used to establish the mice model of asthma referring to our recently published article and relevant literature (24, 25). In detail, the asthma model of mice was established by intraperitoneal injection with 0.1 ml of saline solution containing OVA (0.5 mg/ml) and aluminum hydroxide (2 mg/ml) on days one and seven. From the 15^th^ day, mice were kept in a closed container filled with 2% OVA atomized solution for 21 days, 40 min every day. The mice in the control group were injected with saline solution on the 1st and 7^th^ days and placed in a closed container filled with the saline atomized solution on the 15th day. On days 15–36, the control group and asthma group received 10 ml/kg saline by intragastric administration before 1 hr of each stimulation. The SC group received SC solution at a dose of 0.625 g/kg. The Fer-1 group received 0.8 mg/kg Fer-1 solution through a caudal vein (26). The SC+erastin group received SC solution (0.625 g/kg) and erastin (10 μM). The dose of erastin was according to the previous report (27). Twenty four hours after the last stimulation, blood was gathered from the eyeballs of the mice, and 1 hr later, the sera were separated and stored at -80 °C. Subsequently, mice were euthanized via CO_2_ inhalation. The left lung was acquired from the mice, partially immobilized, and the remaining tissues were stored in a -80 °C refrigerator.

***Bronchoalveolar lavage fluid (BALF) collection***

The bronchoalveolar lavage was performed with 1 ml precooled sterile PBS solution and repeated 3 times. BALF was collected with approximately 80% and centrifuged for 5 min at 500 g/min, 4 °C, and the supernatant was gathered for detecting the cytokines.

***Cell culture and treatment***

Mouse airway epithelial cells were purchased from the China Center for Type Culture Collection. The cells were cultured in Dulbecco’s modified eagle medium containing 10% fetal bovine serum and penicillin-streptomycin. The cells were cultured in an incubator with 5% carbon dioxide at 37 °C. When the cells came up to 80–90% confluence, trypsin was used to digest adherent cells for subsequent experiments. To simulate the airway status of asthma patients, we constructed a cytokine-induced asthma cell model. Mouse airway epithelial cells were stimulated by 10 ng/ml cytokines (interferon-γ (IFN-γ), IL-1β, and TNF-α) for 24 hr for asthma cell model construction.

To study ferroptosis in the asthma cell model, the cells were randomly divided into three groups (n=3), including the control group, model group, and model+Fer-1 group. To investigate whether SC improves ferroptosis in the asthma cell model, cells were divided into three other groups (n=3), including control, model, and model+SC. The dose of Fer-1 (2 μM) was in accordance with the previous report (26). The control group was treated with PBS. Then, cells were collected for subsequent experiments.

***Cell counting Kit-8 (CCK8) assay***

The cytotoxicity of SC was determined via CCK-8 assay. Cells were inoculated in 96-well plates with 1×10^4^ cells/ml. After 24 hr, SC was used to stimulate the cells in final concentrations of 0.1 mg/ml, 0.01 mg/ml, 0.001 mg/ml, and 0.0001 mg/ml CCK-8 solution (Beyotime, China) was added after culturing for 12, 24, 36, or 48 hr, and the absorbance was measured by using a spectrophotometer (BD Biosciences, USA) at 450 nm.

***Quantitative reverse transcription polymerase chain reaction (qRT-PCR)***

Total RNA was obtained from the cells and lung tissues and the concentration was measured by a Nanodrop ND-1000 spectrophotometer (Tiangen Biotech Co., Ltd.). Reverse transcription was performed with the Maxima First Strand cDNA Synthesis Kit (Promega, USA) for RT-PCR. Ferroptosis-related genes were quantified using a Power SYBR Green real-time PCR kit (Qiagen, Hilden, Germany) in an ABI Q6 real-time PCR machine (Applied Biosystems Inc., USA). GAPDH was used as an internal reference gene. The relative mRNA expression levels of FTH1, GPX4, and GAPDH were computed by the 2^−ΔΔCt^ method. The sequences of all gene primers were shown in Table S1.

***Western blot (WB) analysis***

The lung tissues and cells were lysed with RIPA buffer (Beyotime, China) and sonicated. A protein assay kit was applied to measure the concentration of total proteins. Equal protein was added in a 10% SDS gel and then transferred to polyvinylidene fluoride membranes. Afterward, membranes were blocked in 5% milk for 1 hr. Primary antibodies against FTH1 (1:1000; Sigma, CA, USA), GPX4 (1:1000; Sigma, CA, USA), and GAPDH (1:1000; Sigma, CA, USA) were used to incubate membranes overnight at 4 °C. Membranes were washed and incubated with goat anti-rabbit IgG-horseradish peroxidase secondary antibody (1:1000; Sigma, CA, USA) for 2 hr. An enhanced chemiluminescence reagent was used to visualize the bands and the bands were analyzed using Bio-rad Image Lab (Software 5.2.1).

***Hematoxylin-eosin (HE) staining***

The lung tissues of each group of mice were selected for HE staining. Briefly, formalin-fixed lung tissues were fixed in 10% buffered formaldehyde (Shanghai Biotechnology Co. Ltd, Shanghai, China), dehydrated, and embedded in paraffin, cell nuclei were counterstained with hematoxylin (Shanghai Biotechnology Co. Ltd, Shanghai, China) and cytoplasm was stained with eosin. Finally, the sections were dehydrated in gradient ethanol and diaphanizated, and the slides were sealed with neutral balsam. The pathological changes in lung tissues were observed using a light microscope (LEICA DM500, Wetzlar, Germany).

***Enzyme-linked immunosorbent assay (ELISA)***

The levels of IL-1β and TNF-α were detected in the BALF of mice and the supernatant of airway epithelial cells by ELISA kits (Boster Biological Technology Co.Ltd, USA) in accordance with the instructions from the manufacturer. The absorbance was measured at 450 nm using a Microplate Reader (Thermo Scientific, NY, USA).

***Evaluation of malondialdehyde (MDA) and reactive ferrous iron (Fe*** ^2+^ ***)***

The MDA Assay kit and Iron Assay kit (Beyotime, China) were used to measure the concentrations of MDA and Fe^2+^ in the lung tissues and airway epithelial cells, respectively. The absorbance of MDA and Fe^2+^ was detected at 450 nm by a spectrophotometer (Thermo Fisher Scientific, MA, USA).

***Measurement of reactive oxygen species (ROS)***

To assess the production of ROS in the airway epithelial cells of mice, cells were incubated in 10 μmol/L 2,7-dichlorofluorescein diacetate (DCFH-DA) (Sigma-Aldrich, USA) in an incubator at 37 °C for 30 min. The sample was mixed upside down every 3–5 min so that the probe was completely in contact with the cells. Then, cells were washed with serum-free cell culture medium 3 times, and 4’,6-diamidino-2-phenylindole (Thermo Fisher, USA) was used to stain the cell nucleus. Laser confocal microscopy (Nikon, Japan) was used to observe the expression of ROS.

***Statistical analysis***

Data were analyzed by SPSS 24.0 and presented as mean±SD. One-way analysis of variance followed by Tukey’s test was used to calculate the difference between more than two groups. The difference is significant with *P*<0.05 as the inclusion criteria.

## Results

***Fer-1 attenuates inflammation of asthma mice***

**Figure 1:**
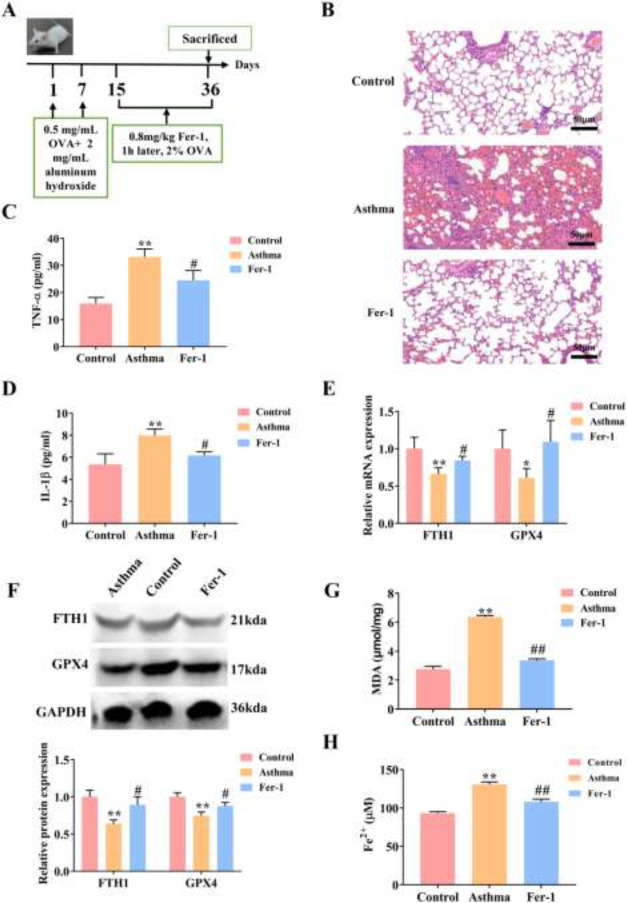
Ferroptosis occurs during asthma and Fer-1 attenuates inflammation of asthma model mice

To study the ameliorative effect of ferroptosis on asthma, we established the asthma mice model and treated the mice with Fer-1 (Figure 1A). The pathological changes of the lung tissues were detected using H&E staining. As shown in Figure 1B, HE staining showed that infiltrated inflammatory cells and mucus secretion increased in the mice model of asthma relative to the control group. Fer-1 reduced the infiltration of inflammatory cells and mucus secretion induced by OVA. ELISA analysis showed that the levels of TNF-α and IL-1β were significantly increased in the BALF of asthmatic mice compared with the control mice (*P*<0.01) (Figures 1C and 1D), while Fer-1 remarkably suppressed the secretion of TNF-α and IL-1β (*P*<0.05). qRT-PCR results demonstrated that the mRNA expressions of FTH1 (*P*<0.01) and GPX4 (*P*<0.05) were obviously decreased in asthmatic mice. However, Fer-1 remarkably facilitated FTH1 and GPX4 mRNA expression (*P*<0.05) (Figure 1E). In parallel, WB analysis revealed that the protein expressions of FTH1 and GPX4 were decreased in asthmatic mice (*P*<0.01), while increasing in asthmatic mice under Fer-1 treatment (*P*<0.05) (Figure 1F). In addition, the levels of MDA and Fe^2+ ^were increased in asthmatic mice compared with the control mice (*P*<0.01), while Fer-1 significantly limited MDA and Fe^2+ ^accumulation (*P*<0.01) (Figures 1G and 1H). These findings suggested that ferroptosis occurred in asthma, and Fer-1 relieved inflammation in asthma mice.

***SC improves asthma by inhibiting ferroptosis in vivo***

**Figure 2:**
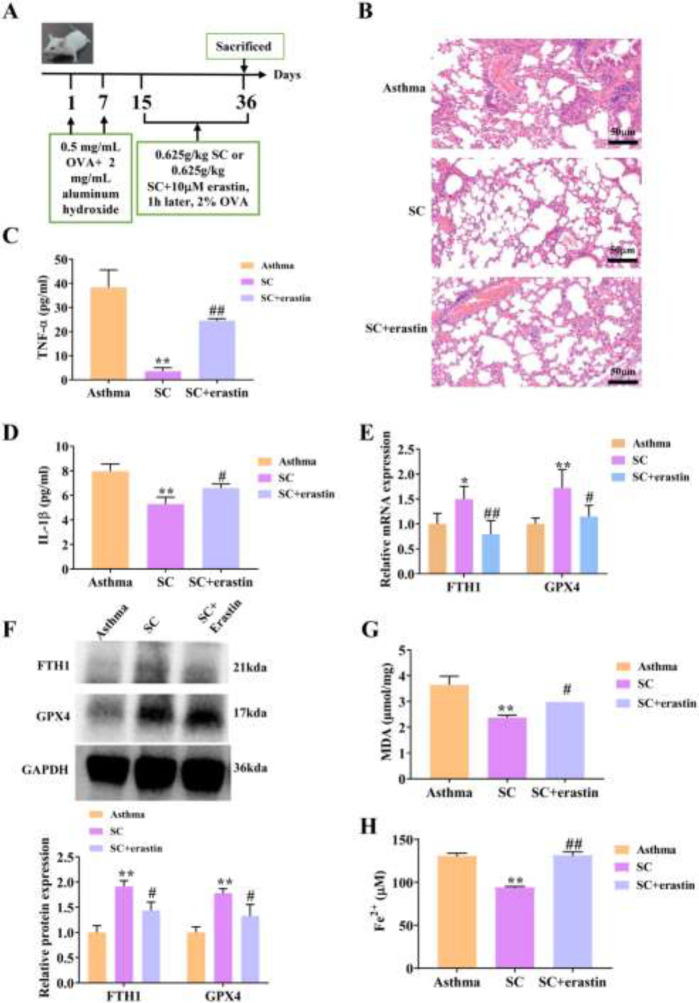
SC ameliorates asthma by regulating ferroptosis

We have previously found that SC can improve asthma inflammation, and we further investigated whether ferroptosis is involved in the ameliorative effect of SC on asthma. We established the asthma mice model and treated the mice with SC (Figure 2A). HE staining showed that SC treatment inhibited the infiltration of inflammatory cells and mucus secretion (Figure 2B). ELISA analysis uncovered that SC treatment significantly decreased the levels of TNF-α and IL-1β in BALF of asthmatic mice (*P*<0.05) (Figures 2C and 2D). The mRNA and protein expressions of FTH1 and GPX4 were significantly increased in the lung tissues of the SC group compared with the asthma group (*P*<0.05) (Figures 2E and 2F). Simultaneously, the levels of MDA and Fe^2+^ were dramatically reduced in asthmatic mice treated with SC compared with the asthma group (*P*<0.01) (Figures 2G and 2H). However, the ferroptosis inducer erastin limited the inhibitory effect of SC on ferroptosis in asthmatic mice (*P*<0.05) (Figure 2A-2G), which indicated that SC relieved asthma via inhibiting ferroptosis.

***Ferroptosis increases in the airway epithelial cells stimulated by cytokines***

**Figure 3:**
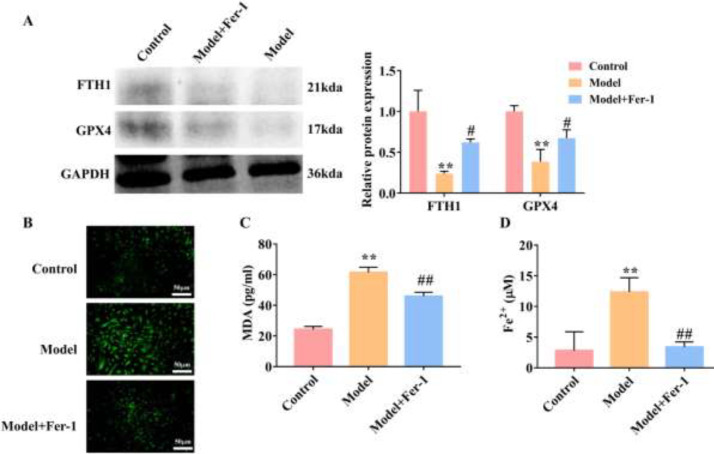
Cytokines stimulate ferroptosis in airway epithelial cells

In order to simulate the airway environment in tissues of patients with asthma, we established the asthma cell model by stimulating the airway epithelial cells with cytokines. As shown in Figure 3A-D, the asthmatic cell model showed a significant decrease in the protein expression of FTH1 and GPX4, and an increase in the accumulation of ROS, MDA, and Fe^2+ ^(*P*<0.01). In comparison with the model group, Fer-1 significantly enhanced the FTH1 and GPX4 expression (*P*<0.05) (Figure 3A). The levels of ROS, MDA, and Fe^2+ ^were obviously reduced in airway epithelial cells stimulated by cytokines under Fer-1 treatment (*P*<0.01) (Figure 3B-3D). These results revealed that ferroptosis occurred in airway epithelial cells stimulated by cytokines.

***SC inhibits ferroptosis of airway epithelial cells induced by cytokines***

**Figure 4:**
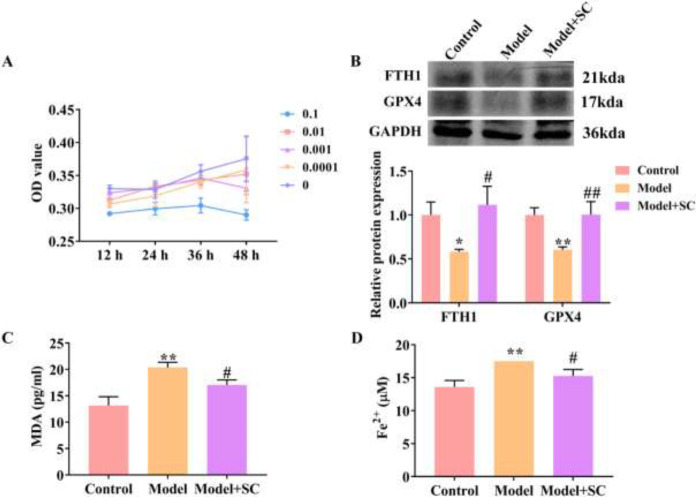
SC inhibits ferroptosis of airway epithelial cells stimulated by cytokines

To explore the mechanism of SC in the development of asthma, we observed the effect of SC in airway epithelial cells stimulated by cytokines. First, the optimal concentration of SC was determined using CCK8, and the result showed that 0.0001 mg/ml SC showed the least effect on cell survival (*P*<0.01) (Figure 4A). SC remarkably facilitated the protein expression of FTH1 (*P*<0.05) and GPX4 (*P*<0.01) (Figure 4B), and suppressed the MDA and Fe^2+^ levels in the asthma cell model (*P*<0.05) (Figures 4C and 4D) compared with the model group. Therefore, SC inhibited the ferroptosis of airway epithelial cells induced by cytokines.

(A) Ovalbumin-induced asthma model. The asthma mice were treated with Fer-1. (B) H&E staining of lung tissues in control, asthma, and Fer-1 groups. Scale bars are 50 μm. (C) Level of TNF-α was determined by ELISA. (D) Level of IL-1β was determined by ELISA. (E) mRNA expression of FTH1 and GPX4 was detected by qRT-PCR. (F) Protein expression of FTH1 and GPX4 was detected by western blot. (G) Level of MDA. (H) Level of Fe^2+^. Fer-1 represents ferrostatin-1. Data are presented as the mean±SD. n=3. One-way ANOVA followed by Tukey’s test was applied to determine the statistical difference. *P<0.05 and **P<0.01 vs control group, #*P*<0.05 and ##*P*<0.01 vs asthma group.

Fer-1 : Ferrostatin-1; FTH1: Ferritin heavy chain 1; GPX4 : Glutathione peroxidase 4; TNF-α: Tumor necrosis factor-α

(A) Ovalbumin-induced asthma model. The asthma mice were treated with SC. (B) H&E staining of lung tissues in asthma, SC, and SC+Erastin groups. Scale bars are 50 μm. (C) Level of TNF-α was determined by ELISA. (D) Level of IL-1β was determined by ELISA. (E) mRNA expression of FTH1 and GPX4 was detected by qRT-PCR. (F) Protein expression of FTH1 and GPX4 was detected by western blot. (G) Level of MDA. (H) Level of Fe^2+^. SC represents scorpio and centipede. Data are presented as the mean±SD. n=3. One-way ANOVA followed by Tukey’s test was applied to determine the statistical difference. *P<0.05 and **P<0.01 vs asthma group, #*P*<0.05 and ##*P*<0.01 vs SC group.

SC: Scorpio and centipede; MDA: Malondialdehyde; FTH1: Ferritin heavy chain 1; GPX4: Glutathione peroxidase 4; TNF-α: Tumor necrosis factor-α

(A) Protein expression of FTH1 and GPX4 was detected by western blot. (B) Level of ROS in airway epithelial cells. Production of ROS was measured using DCFH-DA fluorescent probe. Scale bars are 50 μm. (C) Level of MDA. (D) Level of Fe^2+^. The model represents the asthma cell model. Fer-1 represents ferrostatin-1. Data are presented as the mean±SD. n=3. One-way ANOVA followed by Tukey’s test was applied to determine the statistical difference. *P<0.05 and **P<0.01 vs control group, #*P*<0.05 and ##*P*<0.01 vs model group.

FTH1: Ferritin heavy chain 1; GPX4: Glutathione peroxidase 4; ROS: Reactive oxygen species; DCFH-DA: 2,7-dichlorofluorescein diacetate

(A) Cell viability of airway epithelial cells treated with SC was measured by a CCK-8 assay. n=6. (B) Protein expression of FTH1 and GPX4 was detected by western blot. n=3. (C) Level of MDA. n=3. (D) Level of Fe^2+^. The model represents the asthma cell model. SC represents scorpio and centipede. Data are presented as the mean±SD. n=3. One-way ANOVA followed by Tukey’s test was applied to determine the statistical difference. *P<0.05 and **P<0.01 vs control group, #*P*<0.05 and ##*P*<0.01 vs model group.

SC: Scorpio and centipede; CCK-8: Cell counting Kit-8; MDA: Malondialdehyde; FTH1: Ferritin heavy chain 1; GPX4: Glutathione peroxidase 4

## Discussion

Asthma is a chronic inflammatory disease of the airways, which threatens the lives of people of all ages all over the world. Moreover, the frequency of asthma is rising among children (7). Traditional Chinese medicine was reported to be an effective therapy in asthma treatment (28). A previous study has reported that SC were involved in the treatment of asthma (24). This study aimed to investigate the mechanism by which SC improved asthma. Our study pointed out that the levels of ferroptosis were elevated in asthma mice, and the ferroptosis inhibitor, Fer-1, reduced the levels of inflammation and inhibited ferroptosis in asthma mice and cytokine-induced asthma airway epithelial cells. In parallel, inflammation and ferroptosis were also suppressed by SC in asthma mice and cytokine-induced asthma airway epithelial cells. Furthermore, the combined administration of SC and erastin limited the therapeutic efficacy of SC *in vivo*.

Ferroptosis is a novel type of programmed cell death featured by iron accumulation and lipid peroxidation that results in oxidative stress and cell death. GPX4 is a key regulator of ferroptosis. FTH1 plays an essential part in intracellular iron metabolism. Suppression of GPX4 and FTH1 can promote the occurrence of ferroptosis (29, 30). In our study, we observed a decreased expression of FTH1 and GPX4 in asthma mice, indicating ferroptosis occurred in asthma. Evidence has suggested that ferroptosis plays a crucial part in the development of respiratory diseases, such as asthma (31). Fer-1 ameliorates acute lung injury induced by lipopolysaccharide via restraining ferroptosis (26). Hydrogen sulfide eases particulate matter-induced emphysema and airway inflammation via inhibiting ferroptosis (32). Liproxstatin-1 alleviates neutrophilic asthma in mice via repressing ferroptosis (18). In agreement with these findings, we discovered that SC alleviated asthma by inhibiting ferroptosis *in vivo*. Therefore, ferroptosis may be a new target in the treatment of asthma.

Ample evidence indicates that ferroptosis plays a vital role in inflammation (11). For example, in nonalcoholic steatohepatitis progression, RSL-3, a ferroptosis inducer enhanced the protein levels of proinflammatory cytokines, including TNF-α, IL-1β, and IL-6 (15). Li *et al*. discovered that ferroptosis regulated inflammation in LPS-treated BEAS-2B cells, and panaxydol might alleviate LPS-induced inflammation by repressing ferroptosis (33). IL-6 was demonstrated to induce ferroptosis in bronchial epithelial BEAS-2B cells (34). These results imply that inflammation interacted with ferroptosis. In addition, inflammatory cells promote the dysregulated process of asthma by secreting inflammatory cytokines (35). In this experiment, we found that Fer-1 or SC suppressed the inflammation and ferroptosis of asthma mice, and erastin antagonized the inhibitory effect of SC in asthma mice. Collectively, our study suggested that SC relieved asthma by inhibiting ferroptosis and inflammation.

Bronchial epithelium cell is an important part of the composition of respiratory airways. Normally, bronchial epithelium cells protected the epithelial tissues. While bronchial epithelium cells promoted the production of cytokines and chemokines under-stimulation, therefore resulting in the occurrence of asthma (36). The dysfunction of epithelium cells is relevant to airway inflammation and remodeling (37). The phosphorylation of MAPK and NF-κB was increased in bronchial epithelium cell 16HBE stimulated by LPS, which represents inflammation was enhanced (38). The stimulation of LPS promotes ferroptosis in human bronchial epithelial cells BEAS-2B, while inhibiting ferroptosis reduces cell damage (26). Our findings revealed that cytokines-stimulated ferroptosis in airway epithelial cells and SC suppressed ferroptosis of airway epithelial cells stimulated by cytokines. These results demonstrated that SC alleviated asthma by inhibiting ferroptosis of airway epithelial cells.

## Conclusion

In summary, this study suggested that the crosstalk between ferroptosis and inflammation affected the progress of asthma. SC could mitigate asthma by inhibiting the crosstalk between ferroptosis and inflammation in airway epithelial cells. Our study provides a rational basis for developing the treatment of asthma.

## Authors’ Contributions

Y Z and Y C (Yiren Chen) conceived and designed the experiments. Y W (Yingen Wu) and L Y performed the experiments. H F, Y C (Yanqi Cheng), and Y W (Yuqin Wu) analyzed the data. Y Z, Y C (Yiren Chen), and B T wrote and revised the manuscript. All authors read and approved the final manuscript.

## Data Availability

The datasets used and/or analyzed during the present study are available from the corresponding author upon reasonable request.

## Funding

This work was supported by the Future Plan of Shanghai Hospital of Traditional Chinese Medicine (WL-HBMS-2021001K).

## Etical Approval

This study was approved by the Ethics Committee of the Municipal Hospital of Traditional Chinese Medicine, Shanghai University of Traditional Chinese Medicine, China.

## Conflicts of Interest

The authors declare that there are no conflicts of interest.
